# Fusion‐Brain‐Net: A Novel Deep Fusion Model for Brain Tumor Classification

**DOI:** 10.1002/brb3.70520

**Published:** 2025-05-08

**Authors:** Yasin Kaya, Ezgisu Akat, Serdar Yıldırım

**Affiliations:** ^1^ Department of Artificial Intelligence Engineering Adana Alparslan Turkes Science and Technology University Adana Turkiye; ^2^ Department of Computer Engineering Adana Alparslan Turkes Science and Technology University Adana Turkiye

**Keywords:** brain tumor classification, fusion of CNN, transfer learning

## Abstract

**Problem:**

Brain tumors are among the most prevalent and lethal diseases. Early diagnosis and precise treatment are crucial. However, the manual classification of brain tumors is a laborious and complex task.

**Aim:**

This study aimed to develop a fusion model to address certain limitations of previous works, such as covering diverse image modalities in various datasets.

**Method:**

We presented a hybrid transfer learning model, Fusion‐Brain‐Net, aimed at automatic brain tumor classification. The proposed method included four stages: preprocessing and data augmentation, fusion of deep feature extractions, fine‐tuning, and classification. Integrating the pre‐trained CNN models, VGG16, ResNet50, and MobileNetV2, the model enhanced comprehensive feature extraction while mitigating overfitting issues, improving the model's performance.

**Results:**

The proposed model was rigorously tested and verified on four public datasets: Br35H, Figshare, Nickparvar, and Sartaj. It achieved remarkable accuracy rates of 99.66%, 97.56%, 97.08%, and 93.74%, respectively.

**Conclusion:**

The numerical results highlight that the model should be further investigated for potential use in computer‐aided diagnoses to improve clinical decision‐making.

## Graphical abstract

Using magnetic resonance imaging (MRI) data, we trained a deep fusion model for brain tumor classification. By fusing the pre‐trained CNN models, we enhance comprehensive feature extraction and mitigate over‐fitting issues, improving the model's performance.

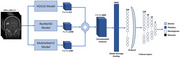

## 1 Introduction

A tumor is an abnormal growth caused by the excessive division and multiplication of cells (Vankdothu et al. 2022, Panyala and Manickam 2024). There are two types of tumors, benign and malignant, which are classified based on their characteristics (Gursoy and Kaya 2024, Togacar et al. 2020, Kumar et al. 2021). Benign tumors remain in their original location and do not spread to other body parts. Typically, patients with benign tumors might continue their lives normally. Malignant tumors are characterized by cells that grow uncontrollably and can spread to nearby or distant areas (Togacar et al. 2020). If the tumors develop in the brain, which is the control center of the nervous system that controls our behavior, they may be fatal (Lu et al. 2021, Togacar et al. 2021). Brain tumors rank second in death rate after cardiovascular diseases in today's world (Alhassan and Zainon 2021). There are various types of brain tumors, depending on the tumor's shape, texture, and location (Abdelaziz Ismael et al. 2020). Three common types of brain tumors are gliomas, pituitary, and meningioma (Kesav and Jibukumar 2022). Gliomas are the most common type of primary brain tumor that arises from glial cells, particularly astrocytes, and are detected in the cerebral hemispheres. Meningiomas are typically non‐cancerous growths that originate from the meninges (Abdelaziz Ismael et al. 2020, Kesav and Jibukumar 2022, Sharma et al. 2023). Pituitary tumors occur in the pituitary gland located in the brain (Rehman et al. 2020). Early and accurate diagnosis is crucial for the patient's wellbeing (Vankdothu et al. 2022, Saurav et al. 2023). Medical professionals utilize various techniques to diagnose tumor type and stage for precise and effective treatment. The use of biopsy, positron emission tomography (PET), computed tomography (CT), and magnetic resonance imaging (MRI) techniques are widespread in the medical field (Vankdothu et al. 2022, Togacar et al. 2021, Kesav and Jibukumar 2022, Ayadi et al. 2021). MRI is a more commonly used medical imaging technique due to its high resolution and non‐invasive nature (Kesav and Jibukumar 2022, Ayadi et al. 2021, Singh et al. 2021).

Manual tumor diagnosis and type classification are time‐consuming and tedious processes, requiring doctors’ and radiologists’ expertise. Thanks to the developments in computer technology, the diagnosis and classification of brain tumors can be made automatically (Abdelaziz Ismael et al. 2020, Sharma et al. 2023, Cinar and Yildirim 2020, Gupta et al. 2024). A computer‐assisted diagnosis (CAD) system based on artificial intelligence has recently been used to classify and diagnose tumors (Abdelaziz Ismael et al. 2020, Husham et al. 2020, Bodapati et al. 2021). A typical CAD system has three steps: lesion segmentation, tumor characteristics extraction, and machine learning methods for classification (Abdelaziz Ismael et al. 2020). Deep learning (DL), a subfield of ML, eliminates the need for hand‐crafted features. Additionally, DL has successfully minimized the gap between human and computer vision for pattern recognition and can outperform traditional techniques (Ayadi et al. 2021, Gursoy and Kaya 2025, Gupta et al. 2023, Topuz and Kaya 2025).

Several DL and ML models have been proposed for brain tumor classification in the literature (Rajinikanth et al. 2022). However, many studies have reported certain limitations. Ismael et al. (2020) tested their model using a small dataset with three classes. Evaluating the model's performance and conducting experiments on larger datasets with more tumor types is important. Brunese et al. (2020) stated that although ML algorithms can solve classification tasks without prior domain knowledge, a minimum deviation from the learned behavior can cause misclassification of instances not in the training dataset. In Bodapati et al. (2021), the authors specified that using two pre‐trained models for feature extraction has some limitations and that increasing the number of models to extract deep features might improve the model's performance. Another challenge is that the various forms of MRI and the different contrast levels make this problem complex (Khan et al. 2024). Brain tumors differ in size, location, growth patterns, and molecular features (Gursoy and Kaya 2024).

To handle the limitations of the recent works, Kaur and Mahajan (Kaur and Mahajan 2025) presented a ResNet152‐based DL model to identify brain tumors. The authors employed dimensionality reduction, feature extraction through ResNet152, and SVM as a classifier. They combined computationally complex models and architectures.

In Adrian et al. (2021), Kaya and Gursoy (2023), and He et al. (2021), the authors show that the fusion method improves classification accuracy and minimizes classification errors. The impressive performance of fusion models in image classification led us to conduct this study. Thus, the study aims to develop a fusion model to address the above limitations.

In this study, we proposed a novel hybrid transfer learning (TL) model for identifying brain tumors. The proposed model combines three pre‐trained convolutional neural networks (CNNs)—VGG16, ResNet50, and MobileNetV2—as feature extractors, followed by a fine‐tuning process. By utilizing these pre‐trained models for fusion, we achieved effective results even with smaller datasets and reduced training times (Remzan et al. 2023). The data augmentation technique was also employed on the datasets to increase the variety of the data and the model's performance. The proposed model was trained on four publicly available datasets, and successful results were attained.

The main contributions of our study can be summarized as follows:

1. A new deep transfer learning model was proposed to classify brain tumors by fusing features obtained from ResNet50, VGG16, and MobileNetV2.
2. The model's performance was improved by integrating a fine‐tuning process.
3. Experiments were performed on four public datasets, and a comparative performance analysis was conducted.
4. The proposed model achieved accuracy rates of 99.66%, 97.56%, 97.08%, and 93.74% on the Br35H, Figshare, Nickparvar, and Sartaj datasets, respectively.

The rest of the paper is organized as follows: Section 2 presents related works. Section 3 introduces information on the materials and methods, containing dataset information, the proposed fused transfer learning model, and its components. Section 4 summarizes and discusses the numerical results obtained from the proposed model. The proposed model and the works in the literature are discussed in Section 5. Lastly, Section 6 presents conclusions and future work.

## 2 Related Work

Deep learning (DL) and machine learning (ML) algorithms have become mainstream in automated medical image analysis (Gursoy and Kaya 2024, Remzan et al. 2023, Sachdeva et al. 2024). Thus, the researchers have focused on utilizing these algorithms to analyze medical images. In Swati et al. (2019), researchers employed a pre‐trained VGG19 model and a block‐wise fine‐tuning to classify the Figshare dataset. The results are compared by changing the number of training datasets at a specific rate. The classification performances of VGG16, AlexNet, and VGG19 models are compared. The results showed that the VGG19 model had the highest accuracy of 94.25%. In another study (Sharma et al. 2023), the authors focused on classifying a dataset in Kaggle using a method combining TL with fine‐tuning. Geometric alterations and changes in tone space methods are used on the dataset. The last layer of the ResNet50 model was removed and replaced with four layers. By training the modified model with the Adam optimizer algorithm, 92% and 100% accuracy values were obtained. In Cinar and Yildirim (2020), the researchers suggested a hybrid model to classify a two‐class dataset. The ResNet50 model was modified by replacing the last five layers with ten new layers. In addition, classification was made in Alexnet, ResNet50, InceptionV3, GoogleNet, and DenseNet201 models. The hybrid model accomplished the highest accuracy rate of 97.01%.

Aurna et al. (2022) presented an ensemble of deep CNN models based on two‐stage feature levels. The proposed model was constructed using five pre‐trained models. The feature‐level ensemble process occurred in two stages. In the first stage, the best ensemble models were determined by combining scratched CNN, EfficientNetB0, and ResNet50 from six models. In the second stage, selected models from the initial phase, ResNet50 + EfficientNetB0 and ResNet50 + scratched CNN, were utilized. After adding a dense layer, the two models were concatenated, with a dropout applied at a ratio of 0.5. The principal component analysis (PCA) technique was employed to extract informative features by reducing the overall number of features. The proposed model was evaluated on four datasets: Figshare, Sartaj, a 4‐class dataset, and a merged dataset. Data augmentation techniques were used for the datasets. The accuracies of Figshare, Sartaj, the 4‐class dataset, and the merged dataset were 99.67%, 98.16%, 99.76%, and 98.96%, respectively. Amran et al. (2022) utilized the GoogleNet model to classify the Br35H dataset. The last five layers of the GoogleNet model were replaced with 14 new layers, and the data augmentation technique was also implemented. After comparing the accuracies of different pre‐trained models, the authors reported that the suggested model achieved an accuracy rate of 99.51%.

Kabir Anaraki et al. (2019) conducted a classification study on the Figshare dataset and glioma grades. Normalization and data augmentation techniques were applied to the datasets. A new model employed a CNN and a genetic algorithm (GA). The bagging method was also utilized to reduce the error rate. Two separate models were set up for two different datasets. One model, with five convolutional layers, max pooling, and one fully connected layer, was established to classify glioma grades. In contrast, another model with six convolutional layers, max pooling, and one fully connected layer was created to classify the Figshare dataset. The dropout technique was employed in both models. An accuracy of 90.9% was achieved for the glioma grades dataset, and 94.2% was achieved for the Figshare dataset. Vankdothu et al. (2022) suggested a combination of convolutional neural networks (CNN) and long short‐term memory (LSTM) models to train a dataset with four classes. The accuracy values obtained from CNN, recurrent neural networks (RNNs), and CNN‐LSTM models are 89.39%, 90.02%, and 92%, respectively. Mondal and Shrivastava (Mondal and Shrivastava 2022) proposed the BMRI‐Net model, which consists of three steps. In the first step, there is a single layer of convolution followed by batch normalization and an activation layer. The second step consists of seven convolutional blocks, with a max‐pool layer in the final step. Due to the bias shift effect and neuron death, the parametric flatten‐p mish (PFpM) activation function is used instead of ReLU. The Br35H and Figshare datasets were employed to test the model. Data augmentation and contrast enhancement techniques were applied to the images. To evaluate the effectiveness of the proposed model, it was tested with different activation functions and pre‐trained models. The model achieved the highest accuracy of 99.00% on Br35H and 99.57% on Figshare.

Arumugam et al. (2024) attempted to detect brain tumors using an MLP model. The optimization algorithm employed a crossover search agent to determine the optimal weights and biases, thereby minimizing the MLP classification error. A dataset with two classes was created by combining three datasets, one of which was Nickparvar. A bilateral filter (BF) was utilized to remove image noise, while a gray‐level co‐occurrence matrix (GLCM) acted as a feature extractor. The CSA‐MLP model achieved the highest accuracy rate of 98.56% compared to other models. Remzan et al. (2023) proposed an ensemble model for classifying the Nickparvar dataset. The BF was used to preserve edges while smoothing images. Additionally, gamma correction was applied to enhance image quality. Seven pre‐trained CNNs and various machine‐learning algorithms were employed to develop the model. By comparing pre‐trained models with ML algorithms, a novel model was formed by ensembling EfficientNetB1, ResNet50, VGG19 pre‐trained models, and an MLP. The proposed model achieved an accuracy rate of 96.67%.

Dutta et al. (2024) proposed an attention‐based residual multiscale CNN, ARM‐Net, for multiclass brain tumor classification. ARM‐Net achieved an accuracy of 96.64% on the MBTD dataset. In another study (Mehrotra et al. 2024), the authors introduced a hybrid model featuring discrete wavelet transform, deep convolutional network, and machine learning methods for brain tumor identification. They utilized a Kaggle brain tumor MR images dataset and attained an AUC of 99.5%. In Kaur and Mahajan (2025), the ResNet152‐based deep learning model was proposed to detect brain tumors in MR images. The model was evaluated on the Br35 dataset, achieving an accuracy rate of 98.53% through dimensionality reduction, feature extraction via ResNet152, and SVM as a classifier. Subba and Sunaniya (2025) presented an attention‐based GoogLeNet‐style CNN model for brain tumor classification. They assessed their model on the Figshare dataset and achieved an overall accuracy of 97.62%. In another study, which uses a combination of ML and DL methods, Khan et al. (2024) proposed a model that integrates DenseNet169 as a feature extractor with RF, SVM, and XGBoost as classifiers. They evaluated their model on the Figshare dataset and achieved an overall classification accuracy of 95.10%. Gursoy and Kaya (2024) proposed a Graph CNN‐based model to identify brain tumors. They evaluated their model by merging two publicly available datasets for broader representation, achieving an average accuracy rate of 93.68%. Zebari et al. (2024) proposed a fusion deep learning model for brain tumor classification in MR images. They assessed their model on a public dataset, attaining an accuracy rate of 98.98%.

Previous studies have shown that deep learning architectures are frequently employed to classify brain tumors. Some studies have used various techniques, such as data augmentation, feature extraction, and filtering, to perform preprocessing. One significant challenge of these works is that they evaluated their models on small datasets. However, they reported that these studies yielded compelling results.

## 3 Material and Methods

**FIGURE 1:**
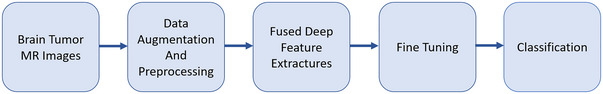
The block diagram of the suggested approach.

The proposed model, FusionBrainNet, is a hybrid transfer learning‐based approach that comprises four steps: preprocessing and data augmentation, fused deep feature extraction, fine‐tuning, and classification, as shown in Figure 1. In the preprocessing step, the images in the dataset are resized to fit the model input. The datasets are divided into training and testing sets. In the data augmentation (DA) step, several morphological DA techniques are applied to the images to enhance model training. Following this, the proposed model is trained and fine‐tuned. The datasets and detailed descriptions of each step are provided in the following sub‐sections.

### 3.1 Datasets

**FIGURE 2:**
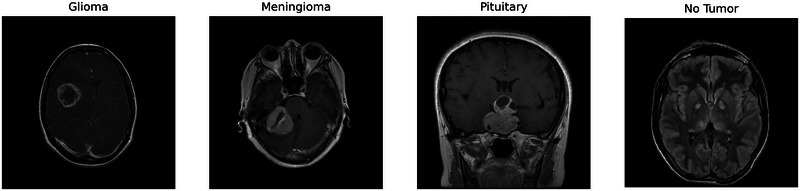
Sample images in the datasets.

In this study, we used four publicly available datasets of MR images to classify brain tumors. The first dataset, the Br35H dataset published by Hamadar, consists of 3000 MR images, 1500 containing tumors and 1500 without tumors (Hamada 2020). The second dataset, obtained from Figshare and comprising 3064 MR images, includes meningioma (708 images), glioma (1,426 images), and pituitary tumor (930 images), as presented by Cheng (2017). The third dataset, created by Nickparvar, encompasses 7023 MR images of brain tumors: 1621 glioma, 1645 meningioma, 1757 pituitary, and 2000 images without tumors (Nickparvar 2021). It was formed by combining three datasets: Figshare, Br35H, and Sartaj. The fourth dataset includes 926 gliomas, 937 meningiomas, 901 pituitary tumors, and 500 images without tumors, totaling 3,264 MRI images of brain tumors created by Dedge (2020). In this study, the databases are referred to as Br35H, Figshare, Nickparvar, and Sartaj, respectively. Figure 2 displays sample images from the Nickparvar dataset.

### 3.2 Preprocessing and Data Augmentation

We randomly split all datasets into 80% for the training dataset and 20% for the test dataset using the same seed. We then separated 20% of the training dataset for the validation dataset across all datasets. We resized all MR images to 224×224×3 and performed normalization to enhance the clarity of the images by adjusting the range of pixel intensity values. Finally, we applied data augmentation techniques such as zooming, vertical and horizontal flipping, brightness adjustments, width and height shifting, and fill mode to the MR images to prevent overfitting and improve generalization performance. The following parameters were utilized for data augmentations: zoom range = 0.15, horizontal flip = true, vertical flip = true, brightness range = [0.05, 1.5], height shift range = 0.05, width shift range = 0.05, and fill mode = “nearest”.

### 3.3 Transfer Learning

Transfer learning (TL) is a crucial deep learning approach (Polat and Gungen 2021), enabling the transfer of weights from a pre‐trained model developed on large datasets for various image classification tasks, allowing their reuse for similar problems (Bodapati et al. 2021). This process results in faster training, reduced data requirements, and improved performance (Polat and Gungen 2021). In this study, we utilized pre‐trained CNN models, specifically VGG16, ResNet50, and MobileNetV2, within the Keras framework for our proposed approach. These models were selected due to their effectiveness in medical image analysis (Gursoy and Kaya 2025, Kaur and Mahajan 2025, Remzan et al. 2023, Swati et al. 2019, Aurna et al. 2022, Zadeh Shirazi et al. 2020).

VGG16, a CNN with 16 layers, achieved an accuracy of 92.7% on the ImageNet dataset, which comprises approximately 14 million images categorized into 1000 classes. It accepts images of 224×224 RGB pixels as input. The convolution layers employed 3×3 pixel filters, and the ReLU activation function was utilized in the hidden layers (Polat and Gungen 2021).

ResNet50 employs 50 convolutional layers trained on the ImageNet dataset. It addresses the vanishing gradient problem common in traditional deep networks by utilizing the shortcut connection method, which bypasses one or more layers. The architecture consists of 5 convolutional blocks, each incorporating a shortcut connection (Kumar et al. 2021, Polat and Gungen 2021). Additionally, bottleneck blocks enhance the training process in ResNet50 (Polat and Gungen 2021).

MobileNetV2 is a deep CNN with 153 layers and an input layer size of 224×224×3. It employs an inverse residual structure connected to the bottleneck layer. Its design is based on two main features: linear bottlenecks between the layers and shortcut connections among the bottlenecks. As a result, the number of parameters is reduced, leading to faster performance and lower latency (Zadeh Shirazi et al. 2020, Maqsood et al. 2022).

### 3.4 The Proposed Fusion‐Based Transfer Learning Model: Fusion‐Brain‐Net

**FIGURE 3:**
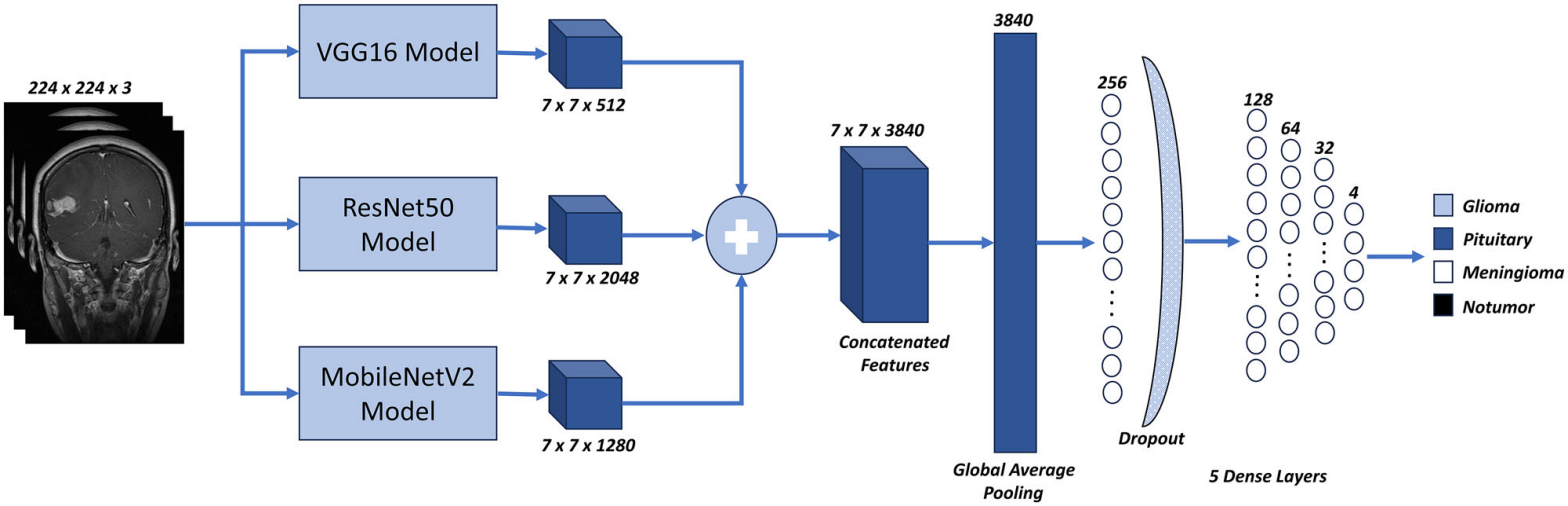
Structural design of proposed architecture.

We fused deep features from the pre‐trained CNN models, specifically VGG16, ResNet50, and MobileNetV2. This approach allowed us to increase the number of informative features to minimize overfitting and enhance the model's performance. We created 3840 features by concatenating 512 from VGG16, 1280 from ResNet50, and 2048 from MobileNetV2, as illustrated in Figure 3. A concatenation layer is employed to merge these features.

We utilized the GlobalAveragePooling2D layer to convert the extracted features into a vector, which served as input for the dense layers. After removing the last layer from all pre‐trained models, we added five dense layers, with the first four having 256, 128, 64, and 32 neurons, respectively, all employing the ReLU activation function. Following the first dense layer, we implemented the dropout layer to prevent overfitting by randomly dropping nodes at a rate of 0.5. In the final dense layer, we applied the sigmoid activation function for binary classification and the softmax activation function for multiclass classification. The number of classes in the dataset determined the number of neurons in the last dense layer.

The mathematical notation of the proposed model is given below:

Let $X\in R^{224\times 224\times 3}$ represent the input image. The model uses three pre‐trained networks (MobileNetV2, VGG16, and ResNet50) as feature extractors. The outputs of these models are concatenated.

Let M represent the MobileNetV2 model (excluding the top layer). The output of MobileNetV2 is calculated as in Equation 1:

(1) $$F_{\text{MobileNetV2}}=M(X)\in R^{h_{1}\times w_{1}\times c_{1}}$$

Let V represent the VGG16 model (excluding the top layer). The output of VGG16 is calculated as in Equation 2:

(2) $$F_{\text{VGG16}}=V(X)\in R^{h_{2}\times w_{2}\times c_{2}}$$

Let R represent the ResNet50 model (excluding the top layer). The output of ResNet50 is calculated as in Equation 3:

(3) $$F_{\text{ResNet50}}=R(X)\in R^{h_{3}\times w_{3}\times c_{3}}$$

The outputs from the three models are concatenated along the depth axis and calculated as in Equation 4:

(4) $$F_{\text{Concat}}=\text{Concat}(F_{\text{VGG16}},F_{\text{MobileNetV2}},F_{\text{ResNet50}})\in R^{h\times w\times c}$$

where:

$$h=\min\left(h_{1},h_{2},h_{3}\right),w=\min\left(w_{1},w_{2},w_{3}\right),c=c_{1}+c_{2}+c_{3}$$

A Global Average Pooling (GAP) operation is applied to reduce the spatial dimensions, as given in Equation 5:

(5) $$F_{\text{GAP}}=\text{GAP}(F_{\text{Concat}})\in R^{c}$$

The feature vector from GAP is passed through a series of fully connected layers, *F*_1_, *F*_2_, *F*_3_, and *F*_4_, as given in Equations 6–9:

(6) $$F_{1}=\text{ReLU}\left(W_{1}F_{\text{GAP}}+b_{1}\right)\;\text{where}\;W_{1}\in R^{256\times c},b_{1}\in R^{256}$$

A dropout operation with a rate of 0.5 is applied to **F**_1_.

(7) $$F_{2}=\text{ReLU}\left(W_{2}F_{1}+b_{2}\right)\;\text{where}\;W_{2}\in R^{128\times 256},b_{2}\in R^{128}$$

(8) $$F_{3}=\text{ReLU}\left(W_{3}F_{2}+b_{3}\right)\;\text{where}\;W_{3}\in R^{64\times 128},b_{3}\in R^{64}$$

(9) $$F_{4}=\text{ReLU}\left(W_{4}F_{3}+b_{4}\right)\;\text{where}\;W_{4}\in R^{32\times 64},b_{4}\in R^{32}$$

The final layer is a softmax classifier with *K* classes (where *K* = Number of Classes), as in Equation 10:

(10) $$\hat{y}=\text{Softmax}\left(W_{5}F_{4}+b_{5}\right)\;\text{where}W_{5}\in R^{K\times 32},b_{5}\in R^{K}$$

VGG16, ResNet50, and MobileNetV2 are widely used deep learning architectures that vary in complexity and efficiency. VGG16, with approximately 138 million parameters and 15.3 GFLOPs, presents a straightforward yet computationally intensive structure. ResNet50, which utilizes residual connections, balances depth and efficiency with 25.6 million parameters and 3.8 GFLOPs. In contrast, MobileNetV2 is optimized for lightweight applications by employing depthwise separable convolutions, resulting in only 3.4 million parameters and 0.3 GFLOPs. In the Fusion‐Brain‐Net model, these architectures function as feature extractors, with only their last two layers unfrozen to fine‐tune high‐level feature representations while preserving their pre‐trained knowledge. This strategy reduces computational overhead compared to full fine‐tuning while allowing domain‐specific refinements.

### 3.5 Fine‐Tuning

We also applied fine‐tuning, a popular transfer learning strategy, to the proposed model (Vrbancic and Podgorelec 2020). Fine‐tuning shifts training from the top to the middle layers and lasts for a few epochs (Can et al. 2021). It enhances pre‐trained models by unfreezing the top layers of the model. In this study, we unfroze the last two layers of all pre‐trained models after a specific training step.

## 4 Experimental Results

### 4.1 Experimental Setup

We conducted all experimental tests on the T4 GPU and 12 GB system RAM provided by the Google Colab platform. For our model training, we set the Adam optimizer's learning rate to 0.001, the epoch to 100, and the batch size to 32. We employed accuracy, precision, recall, and F1‐score metrics in the study to assess the proposed model's performance, as given in Equations (11), (12), (13), and 14:

(11) $$\mathrm{Accuracy}=\frac{TP+TN}{TP+FN+FP+TN}$$

(12) $$\mathrm{Precision}=\frac{TP}{TP+FP}$$

(13) $$\mathrm{Recall}=\frac{TP}{TP+FP}$$

(14) $$F1-\mathrm{score}=\frac{2\cdot \mathrm{Prec}\mathrm{ision}\cdot \mathrm{Reca}\mathrm{ll}}{\mathrm{Prec}\mathrm{ision}+\mathrm{Reca}\mathrm{ll}}$$

where TP represents the number of true positives, TN represents the number of true negatives, FP represents the number of false positives, and FN represents the number of false negatives. Accuracy is the ratio of correctly predicted instances to the total instances in a dataset. Precision represents the proportion of correctly predicted positive cases among all cases predicted as positive. Recall is the proportion of correctly predicted positive cases among all actual positive cases. The F1‐score is the harmonic mean of precision and recall, balancing both metrics in a single value.

**FIGURE 4:**
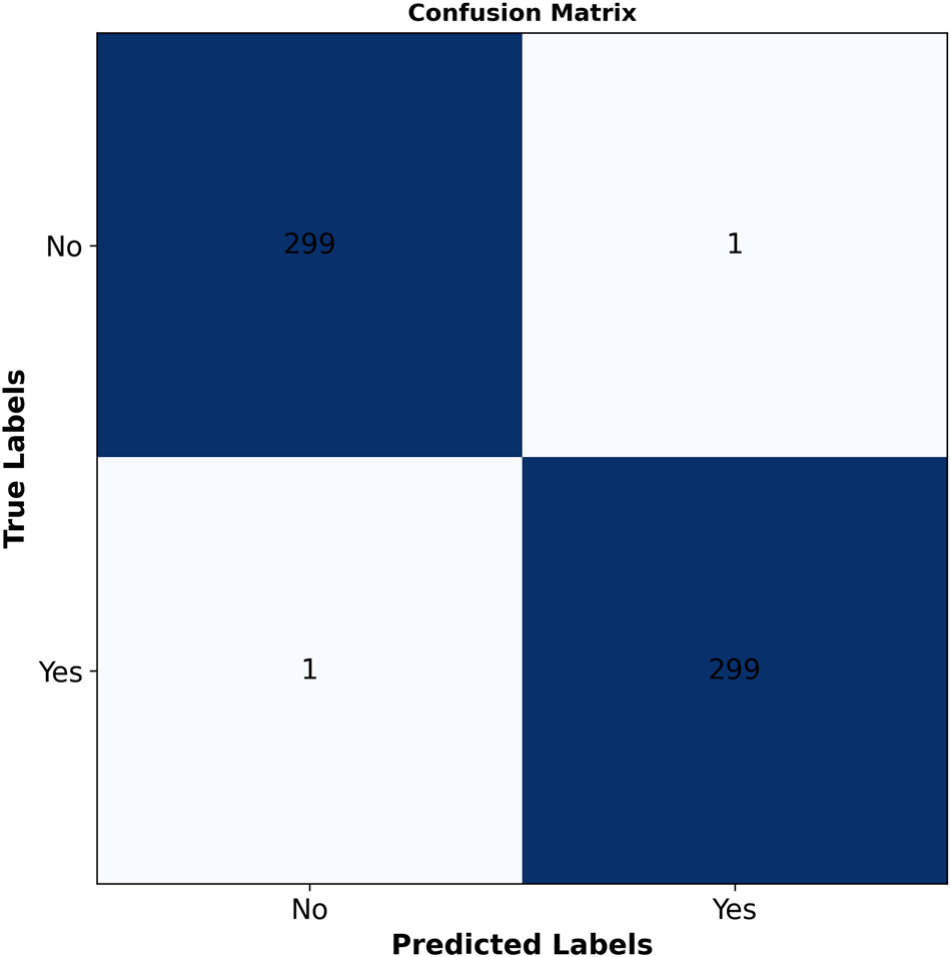
The confusion matrix for the proposed fine‐tuned model on the Br35H dataset.

**FIGURE 5:**
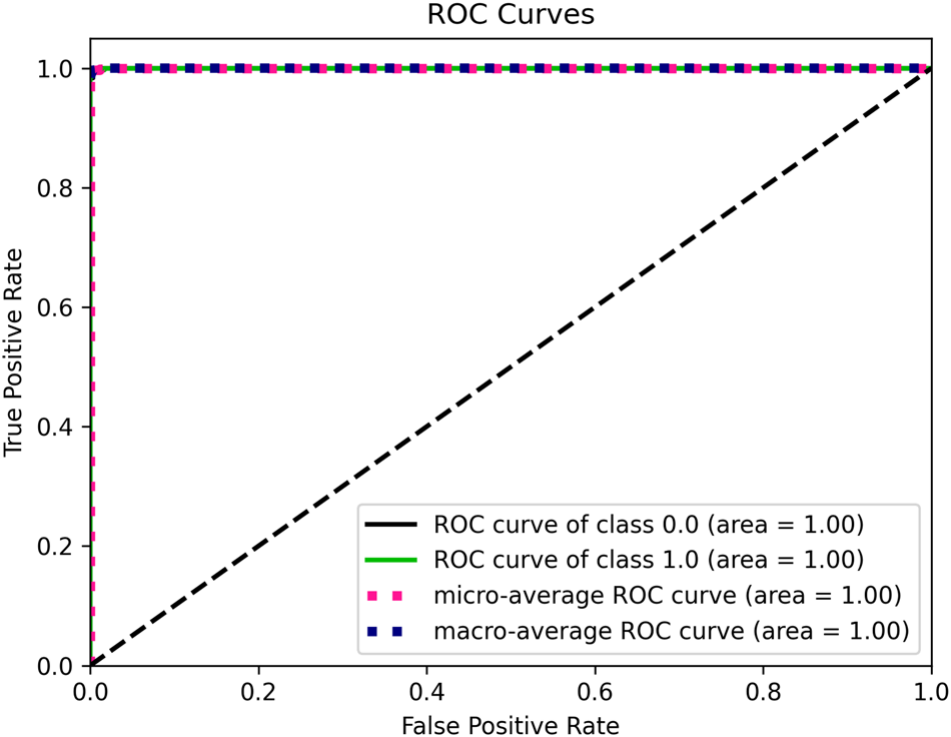
The ROC curve obtained from the Br35H dataset.

**FIGURE 6:**
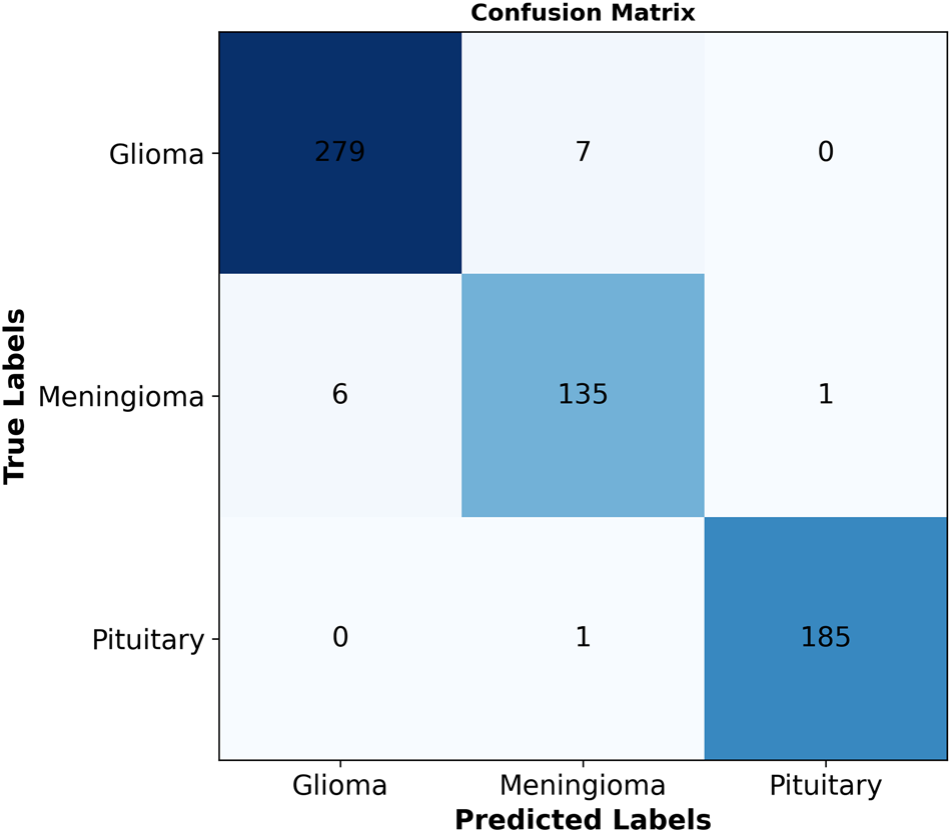
The confusion matrix for the proposed fine‐tuned model on the figshare dataset.

**FIGURE 7:**
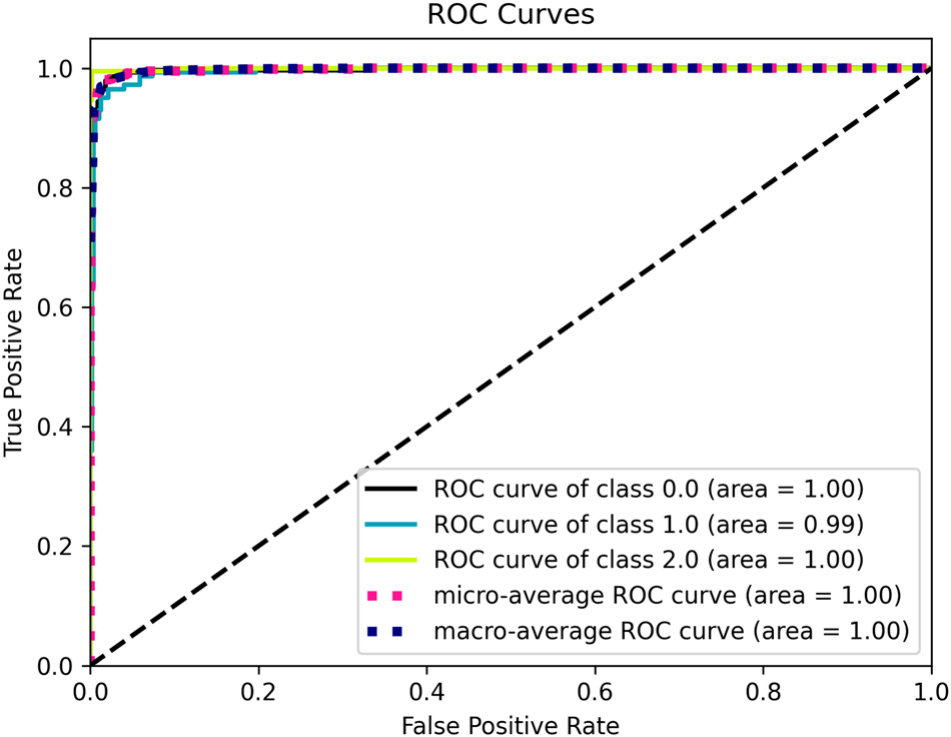
The ROC curve obtained from the Figshare dataset.

**FIGURE 8:**
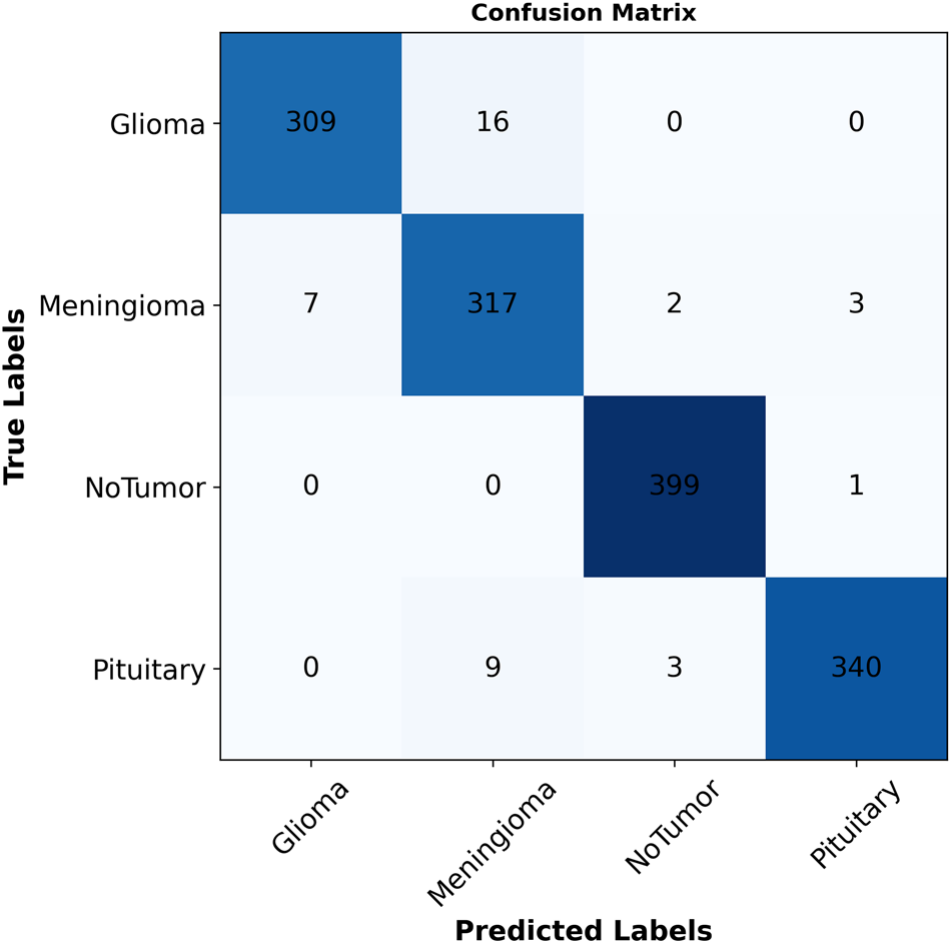
The confusion matrix for the proposed fine‐tuned model on the nickparvar dataset.

**FIGURE 9:**
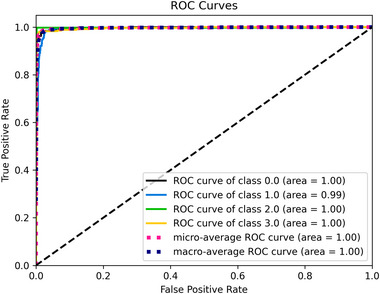
The ROC curve obtained from the nickparvar dataset.

**FIGURE 10:**
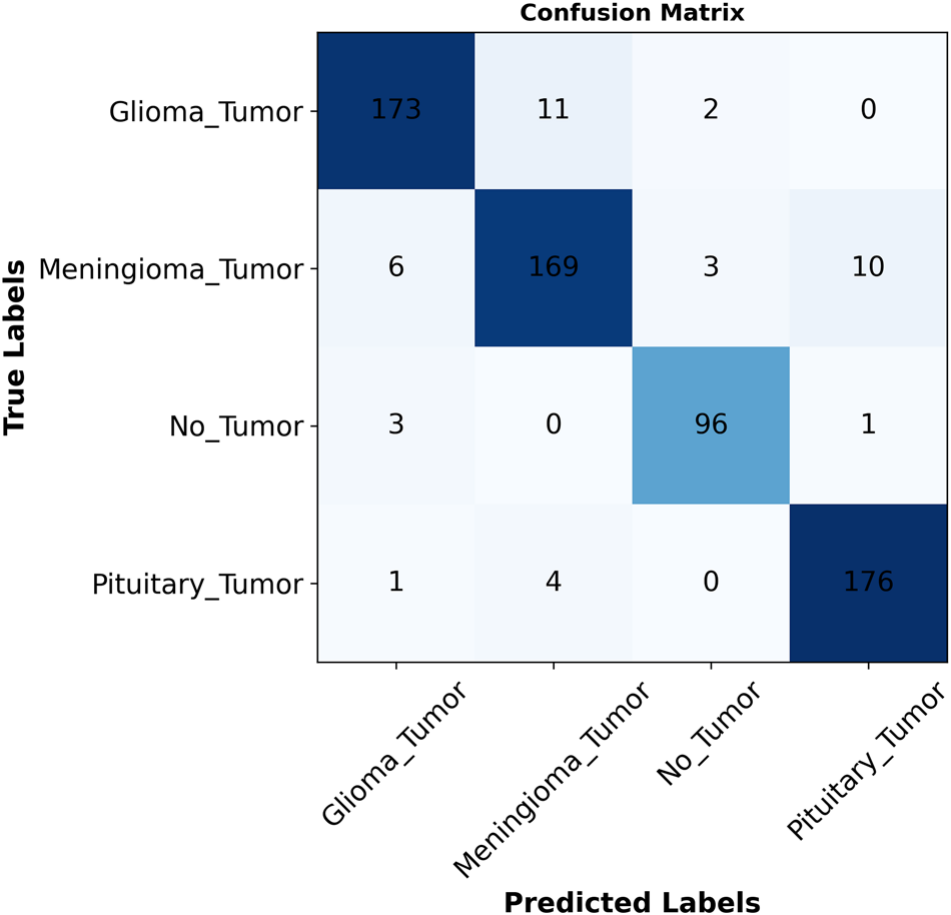
The confusion matrix calculated on the sartaj dataset.

**FIGURE 11:**
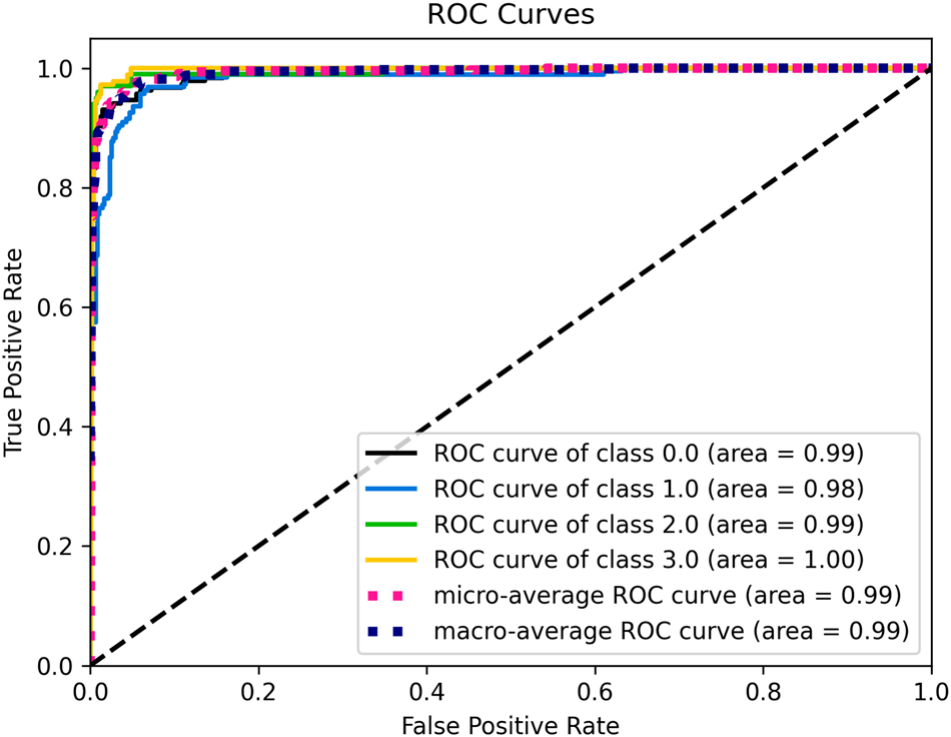
The ROC curve obtained from the sartaj dataset.

We conducted experimental tests across three categories. In the first, we independently employed pre‐trained models. Next, we evaluated the proposed model by combining the extracted features from these models without fine‐tuning. Finally, we fine‐tuned the proposed model as a separate experiment. The experiments utilized four datasets: Br35H, Figshare, Nickparvar, and Sartaj. Tables 1, 2, 4, and 3 illustrate the results of the experiments using evaluation metrics: accuracy, precision, recall, and F1‐score. Figures 4, 6, 8, and 10 present confusion matrices, with each row representing the actual class and each column representing the predicted class. Figures 5, 7, 9, and 11 display Receiver Operating Characteristic (ROC) curves, which plot the false positive rate on the X‐axis against the true positive rate on the Y‐axis. The area under the ROC curve is known as the AUC, which ranges from 0 to 1, indicating the classification performance of a model. A value closer to 1 signifies a higher level of success in classification performance (Polat and Gungen 2021).

**TABLE 1:** The numerical results on the Br35H dataset.

Method	Accuracy	Precision	Recall	F1‐score
VGG16	0.9883	0.9884	0.9883	0.9883
ResNet50	0.9917	0.9917	0.9917	0.9917
MobileNetV2	0.9650	0.9650	0.9650	0.9650
Fusion‐Brain‐Net	0.9900	0.9900	0.9900	0.9900
Fusion‐Brain‐Net with fine‐tuning	**0.9967**	**0.9967**	**0.9967**	**0.9967**

**TABLE 2:** The numerical results on the nickparvar dataset.

Method	Accuracy	Precision	Recall	F1‐score
VGG16	0.9488	0.9516	0.9462	0.9466
ResNet50	0.9467	0.9453	0.9444	0.9445
MobileNetV2	0.9324	0.9309	0.9294	0.9297
Fusion‐Brain‐Net	0.9673	0.9669	0.9660	0.9660
Fusion‐Brain‐Net with fine‐tuning	**0.9708**	**0.9702**	**0.9694**	**0.9696**

**TABLE 3:** The numerical results on the sartaj dataset.

Method	Accuracy	Precision	Recall	F1‐score
VGG16	0.9160	0.9176	0.9205	0.9188
ResNet50	0.9145	0.9195	0.9239	0.9213
MobileNetV2	0.8809	0.8808	0.8805	0.8786
Fusion‐Brain‐Net	0.9130	0.9053	0.9188	0.9104
Fusion‐Brain‐Net with fine‐tuning	**0.9374**	**0.9389**	**0.9404**	**0.9395**

### 4.2 Numerical Results

In this section, we present the numerical results obtained from experimental tests. Table 1 presents the numerical results for the Br35H dataset. The MobileNetV2 model achieved the lowest performance, obtaining accuracy, precision, recall, and F1‐score values of 0.9650, 0.9650, 0.9650, and 0.9650, respectively. In contrast, the proposed model achieved a value of 0.99 across all metrics. It is evident that fine‐tuning slightly improved all metrics, resulting in a value of 0.9967. There was approximately a 3% accuracy increase between MobileNetV2 and the proposed fine‐tuned model.

Figure 4 illustrates the confusion matrix for the Br35H dataset. While 299 *No* and 299 *Yes* were accurately classified, 1 *No* and 1 *Yes* were misclassified using the fine‐tuned fused model. Consequently, the correct classification rates for *Yes* and *No* images were 100%. Figure 5 depicts the ROC curve for the Br35H dataset. The AUC was found to be one at the end of the test.

Table 2 compares the results of the tested models on the Figshare dataset based on performance metrics. MobileNetV2 achieved an accuracy of 91.37%, a precision of 90.20%, a recall of 91.31%, and an F1‐score of 90.63%, marking it as the lowest performer. The ResNet50 and VGG16 models performed slightly better than MobileNetV2, while the proposed model surpassed the separate models. The proposed fine‐tuned model offered the best results, boasting an accuracy of 97.56%, a precision of 97.25%, a recall of 97.36%, and an F1‐score of 97.31%. The fine‐tuned fused model was approximately 4% more accurate than the fused model.

Figure 6 displays a confusion matrix for the classification on the Figshare dataset using the proposed fine‐tuned model. The highest correct classification rate was in the Pituitary, with 99%, followed by Glioma, with 98%, and Meningioma at 95%. There were 279, 135, and 185 correct glioma, Meningioma, and pituitary classifications, respectively. The numbers of misclassifications were 7, 1, and 7 for Glioma, Pituitary, and Meningioma, respectively. Figure 7 shows the ROC curve for the proposed fine‐tuned model on the Figshare dataset, and the AUC value was calculated as 1.

The evaluation results of the proposed fine‐tuned model on the Nickparvar dataset are presented in Table 3. The results of the ResNet50 and VGG16 models were similar, while the MobileNetV2 had the worst results. The proposed model outperformed the separate models by approximately 2%. The proposed fine‐tuned model achieved the highest performance metrics: accuracy, precision, recall, and F1‐score are 97.08%, 97.02%, 96.94%, and 96.96%, respectively.

Figure 8 presents the confusion matrix calculated by the proposed fine‐tuned model on the Nickparvar dataset. As shown in the figure, Glioma had the lowest correct classification rate at 95%, while Meningioma achieved a 96% classification rate. Both

Pituitary and notumor attained the highest accuracy rate of 100%. The numbers of accurate classifications for glioma, meningioma, notumor, and pituitary were 309, 317, 399, and 400, respectively, with incorrect classifications of 16, 12, 1, and 12 for glioma, meningioma, notumor, and pituitary. Figure 9 demonstrates the ROC curve from this classification experiment, with an AUC value calculated as 1.

Table 4 presents the experimental results on the sartaj dataset. MobileNetV2 recorded the lowest evaluation metrics, with accuracy, precision, recall, and F1‐scores of 88.09%, 88.08%, 88.05%, and 87.96%, respectively. VGG16, ResNet50, and the proposed models attained similar accuracy rates. The proposed fine‐tuned model provided approximately 5% higher accuracy than MobileNetV2 and around 2% higher than the other models.

**TABLE 4:** The numerical results on the figshare dataset.

Method	Accuracy	Precision	Recall	F1‐score
VGG16	0.9251	0.9162	0.9161	0.9159
ResNet50	0.9300	0.9210	0.9226	0.9217
MobileNetV2	0.9137	0.9020	0.9131	0.9063
Fusion‐Brain‐Net	0.9332	0.9376	0.9137	0.9230
Fusion‐Brain‐Net with fine‐tuning	**0.9756**	**0.9725**	**0.9736**	**0.9731**

Figure 10 illustrates the confusion matrix for the sartaj dataset obtained from the proposed fine‐tuned model. The highest classification accuracy was for Pituitary Tumor at 97%, followed by No Tumor at 96%. The lowest classification rate was for Meningioma Tumor at 90%, followed by Glioma Tumor at 93%. The number of correct classifications for Glioma Tumor, Meningioma Tumor, No Tumor, and Pituitary Tumor were 173, 169, 96, and 176, respectively. The misclassifications for Glioma Tumor, Meningioma Tumor, No Tumor, and Pituitary Tumor were 14, 19, 4, and 5, respectively. Figure 11 represents the ROC curve on the same experimental test, and the AUC was 0.99.

Table 5 shows all datasets’ class‐based numerical results for the proposed fine‐tuned model. As can be seen from the table, glomia and pituitary classes attained higher precision scores, while meningoma was the lowest. Additionally, Sartaj and Nickparvar datasets had No Tumor class, which is classified correctly. The experimental tests on the BR35H dataset achieved exceptional results. It might have a positive effect on binary classification.

**TABLE 5:** The class‐based numerical results for fusion‐brain‐net with fine‐tuning on the datasets.

Dataset	Class	Precision	Recall	F1‐Score
Figshare	Glioma Meningioma Pituitary	0.9817 0.8882 0.9788	0.9371 0.9507 0.9946	0.9589 0.9184 0.9867
Sartaj	Glioma Tumor Meningioma Tumor No Tumor Pituitary Tumor	0.9454 0.9185 0.9505 0.9412	0.9301 0.8989 0.9600 0.9724	0.9377 0.9086 0.9552 0.9565
BR35H	No Yes	0.9967 0.9967	0.9967 0.9967	0.9967 0.9967
Nickparvar	Glioma Meningioma Notumor Pituitary	0.9778 0.9269 0.9876 0.9884	0.9508 0.9635 0.9975 0.9659	0.9641 0.9449 0.9925 0.9770

## 5 Discussion

This paper proposes a fine‐tuned fused model designed to classify different types of brain tumors accurately. As illustrated in Tables 1, 2, 4, and 3, the proposed fine‐tuned model achieved the best results across all datasets. The MobileNetV2 model produced the lowest results. In all datasets except for sartaj (0.99), the ROC curve demonstrates an area of 1, indicating excellent classification. Additionally, we noted that while the proposed model was effective on all datasets, the fusion process and fine‐tuning led to increased trainable parameters, resulting in higher computational costs and longer training times. Comparing the 4‐class datasets of sartaj (93.74%) and nickparvar (97.08%), we found that nickparvar has nearly a 4% higher accuracy rate. This difference may be attributed to the sartaj dataset containing 3,264 MR images. In contrast, the nickparvar dataset has 7,023, as a larger data volume significantly enhances model performance in deep learning (Cinar and Yildirim 2020). Furthermore, it was observed that the fusion model was most effective on the Nickparvar dataset. As the complexity of the problem increases, the impact of the fusion process may also amplify.

As demonstrated in Table 6, many studies have been conducted on the Br35H, Figshare, Sartaj, and Nickparvar datasets. Regarding the Figshare dataset, Swati et al. (2019) suggested a model that employs the block‐wise fine‐tuning method on the pre‐trained VGG19 model. Kabir Anaraki et al. (2019) introduced a CNN architecture, which was enhanced using the GA. These two models exhibited lower accuracy values than the proposed model. Concerning the Sartaj dataset, Vankdothu et al. (2022) recommended a hybrid model that combines a CNN with an LSTM, which also achieved a lower accuracy value than the proposed model. Aurna et al. (2022) presented a two‐stage feature ensemble of deep CNN to classify both the Figshare and Sartaj datasets. This study performed the concatenation process twice and utilized three pre‐trained models. Additionally, a scratched CNN model was included in the concatenation process to boost the model's performance. Data augmentation and dropout were implemented, and PCA was also employed. These differences may have contributed to better results than the proposed model's.

**TABLE 6:** The comparison of the proposed model with the latest studies.

Study	Datasets	Methodology	Accuracy
Swati et al. (2019)	Figshare	VGG19	94.82%
Kabir Anaraki et al. (2019)	Figshare	CNN	94.2%
Vankdothu et al. (2022)	Sartaj	CNN, LSTM	92%
Aurna et al. (2022)	Sartaj, figshare	CNN	99.76%, 98.16%
Amran et al. (2022)	Br35H	GoogleNet	99.51%
Mondal and Shrivastava (2022)	Br35H, figshare	CNN	99%, 99.57%
Remzan et al. (2023)	Nickparvar	ResNet50, VGG19, EfficientNetV2B1, MLP	96.67%
Arumugam et al. (2024)	Nickparvar, figshare	CNN, MLP	98.56%
Kaur and Mahajan (Kaur and Mahajan 2025)	Br35	ResNet152 + SVM	98.53%
Subba and Sunaniya (2025)	Figshare	CNN + Softmax	97.62%
Khan et al. (2024)	Figshare	Hybrid‐NET model	95.10%
Zebari et al. (2024)	Kaggle	ResNet50 + CDBN	98.98%
Fusion‐Brain‐Net with fine‐tuning	Br35H, figshare, nickparvar, sartaj	Fusion of CNNs	99.66%, 97.55%, 97.08%, 93.74%

As for the Br35H dataset, Amran et al. (2022) proposed a CNN model based on the GoogLeNet architecture. Mondal and Shrivastava (2022) proposed a new Parametric Flatten‐p Mish activation function‐based deep CNN model trained on the Br35H and Figshare datasets. While the accuracy of the proposed model was higher in the Br35H dataset, it was lower in the Figshare dataset. As for the Nicparvar dataset, in (Remzan et al. 2023), the authors combined features obtained from ResNet50, VGG19, and EfficientNetV2B1 pre‐trained CNN models. Besides, MLP was employed as a classifier. Although using the same dataset, their study may have been less successful due to their utilization of fewer MR images. Arumugam et al. (2024) recommended a novel Crossover smell agent optimized multilayer perceptron CNN model. Their study merged three datasets to train their model on more MR images. Moreover, the dataset was categorized into two classes: tumor and non‐tumor. Since it is easier to classify a two‐class dataset than a four‐class one, deep learning‐based models perform better in larger datasets. Therefore, their model may have been more successful than the proposed model.

In summary, Table 6 presents the results of the most recent studies in the literature and the results of the proposed fine‐tuned model. These studies use models such as trained from scratch CNN models, TL approaches that use pre‐trained models, and ML algorithms. It can be seen from the table that the literature had good results. When compared, the proposed model also has promising results. Thus, the methods developed in this study should be used in computer‐aided diagnosis and implemented in healthcare applications.

### 5.1 Advantages and Drawbacks of the Proposed Model

The advantages of the proposed model can be summarized as follows:

- The Fusion‐Brain‐Net was tested on different datasets and attained high accuracy on all datasets, highlighting the model's effectiveness in brain tumor identification.
- The model uses images that do not need complex preprocessing steps. Thus, the model can be easily implemented in real‐world applications.
- The promising performance obtained on various datasets shows the study's robustness.

One of the biggest challenges of fusion models lies in their combination of diverse models with differing capabilities. As a result, training these models can be pretty demanding. The training datasets should include a larger number of images. If the dataset contains too few images, data augmentation methods should be employed to increase the image count. Evaluating the model with various datasets can enhance its ability to generalize.

## 6 Conclusion

Proper classification of brain tumors is crucial for ensuring patient survival and effective treatment. Therefore, medical professionals and researchers actively explore innovative strategies to address this issue. Nevertheless, existing literature still features gaps that need further exploration. In this research, we introduce a hybrid transfer learning model to classify brain tumors accurately. We employed pre‐trained models, rather than building a CNN from scratch during the fusion process. These models facilitate effective outcomes with smaller datasets and expedited training (Remzan et al. 2023, Polat and Gungen 2021).

The Fusion‐Brain‐Net contains basic preprocessing steps such as normalization and data augmentation. It also includes multi‐branch feature extraction by fusing VGG16, ResNet50, and MobileNetV2 pre‐trained models, enhancing the model's performance and preventing overfitting. The model was fine‐tuned to fit the dataset characteristics better. Finally, we conducted comprehensive experiments to evaluate the performance of the proposed model using four public datasets: Br35H, Figshare, Nickparvar, and Sartaj. Evaluating various datasets, it attained promising results, achieving up to 5% better classification rates in the whole datasets.

This model can be extended for multiclass tumor grading, such as low‐grade vs. high‐grade gliomas. It can also be integrated with multimodal data such as MRI sequences for improved clinical applicability. The proposed model's architecture design parameters and hyperparameters should also be fine‐tuned by a meta‐heuristic algorithm to determine optimal parameters for brain tumor classification.

## Author Contributions

**Yasin Kaya**: conceptualization, methodology, validation, supervision, formal analysis, writing–original draft, writing–review and editing. **Ezgisu Akat**: methodology, data curation, visualization, writing–original draft, writing–review and editing. **Serdar Yıldırım**: formal analysis, supervision, writing–review and editing.

## Ethics Statement

The data for this study was obtained from public databases and can be accessible to everyone. Therefore, this study is exempt from the need for consent to participate.

### Peer Review

The peer review history for this article is available at https://publons.com/publon/10.1002/brb3.70520

## Data Availability

This research uses brain image datasets consisting of Br35H (Hamada 2020), Figshare (Cheng 2017), Nickparvar (Nickparvar 2021), and Sartaj (Dedge 2020), which can publicly accessible.
